# Biomarkers of Residual Disease, Disseminated Tumor Cells, and Metastases in the MMTV-PyMT Breast Cancer Model

**DOI:** 10.1371/journal.pone.0058183

**Published:** 2013-03-08

**Authors:** Christian Franci, Jenny Zhou, Zhaoshi Jiang, Zora Modrusan, Zinaida Good, Erica Jackson, Hosein Kouros-Mehr

**Affiliations:** Research Oncology Department, Genentech, Inc., South San Francisco, California, United States of America; University College London, United Kingdom

## Abstract

Cancer metastases arise in part from disseminated tumor cells originating from the primary tumor and from residual disease persisting after therapy. The identification of biomarkers on micro-metastases, disseminated tumors, and residual disease may yield novel tools for early detection and treatment of these disease states prior to their development into metastases and recurrent tumors. Here we describe the molecular profiling of disseminated tumor cells in lungs, lung metastases, and residual tumor cells in the MMTV-PyMT breast cancer model. MMTV-PyMT mice were bred with actin-GFP mice, and focal hyperplastic lesions from pubertal MMTV-PyMT;actin-GFP mice were orthotopically transplanted into FVB/n mice to track single tumor foci. Tumor-bearing mice were treated with TAC chemotherapy (docetaxel, doxorubicin, cyclophosphamide), and residual and relapsed tumor cells were sorted and profiled by mRNA microarray analysis. Data analysis revealed enrichment of the Jak/Stat pathway, Notch pathway, and epigenetic regulators in residual tumors. Stat1 was significantly up-regulated in a DNA-damage-resistant population of residual tumor cells, and a pre-existing Stat1 sub-population was identified in untreated tumors. Tumor cells from adenomas, carcinomas, lung disseminated tumor cells, and lung metastases were also sorted from MMTV-PyMT transplant mice and profiled by mRNA microarray. Whereas disseminated tumors cells appeared similar to carcinoma cells at the mRNA level, lung metastases were genotypically very different from disseminated cells and primary tumors. Lung metastases were enriched for a number of chromatin-modifying genes and stem cell-associated genes. Histone analysis of H3K4 and H3K9 suggested that lung metastases had been reprogrammed during malignant progression. These data identify novel biomarkers of residual tumor cells and disseminated tumor cells and implicate pathways that may mediate metastasis formation and tumor relapse after therapy.

## Introduction

Metastases are the primary cause of morbidity and mortality in cancer patients. After diagnosis, cancer patients undergo a series of tests to determine their tumor stage, grade, molecular profile, and prognosis. Molecular profiling of a patient’s primary tumor can reveal the likelihood of disease recurrence and metastasis formation [1], [2], [3]. Patients who are at risk of developing metastases at the time of diagnosis may undergo surgery, chemotherapy, radiotherapy, and/or targeted therapy to reduce the likelihood of tumor relapse and metastasis formation [4], [5]. Many patients will nonetheless develop distant metastases in part from residual tumor cells that survived therapy or from disseminated tumor cells and micrometastases that spread from the primary tumor [6], [7]. Residual tumor cells can remain dormant in patients and can give rise to a local tumor recurrence or distant metastases several years after therapy [8], [9], [10]. Similarly, disseminated tumor cells can migrate from the primary tumor to distant organs early during cancer progression [11], [12]. For example, breast cancer patients with no evidence of metastatic disease can have disseminated tumor cells in the bone marrow at the time of diagnosis [9]. These disseminated cells often exhibit fewer genomic aberrations than the primary tumor, suggesting that tumor dissemination can occur early during tumor formation [11]. Nonetheless, disseminated tumor cells often harbor marked genetic heterogeneity, making it difficult to target these populations with targeted therapy [13].

The identification of biomarkers in residual tumors, disseminated tumor cells, and metastases has been challenging because these disease states are difficult to isolate from cancer patients. Studies characterizing patient-derived metastases or residual tumors typically have small sample sizes and often have made contradictory conclusions. For example, some studies of patient-derived metastases have suggested that distant metastases are molecularly distinct from their primary tumors, while other studies indicate that metastases are very similar to their primary tumors [14], [15], [16], [17]. In the laboratory, residual tumors and disseminated tumor cells have been studied in cell culture models, xenograft assays, and genetically engineered mouse models, all of which have limitations in modeling the clinical setting [18]. These studies have identified mechanisms of drug tolerance and dormancy in residual tumors, such as angiogenic dormancy, immunological tolerance, and cellular dormancy [8], [19], [20]. Other studies have identified biomarkers and molecular pathways mediating organ-specific metastatic outgrowth in xenograft models [21], [22], [23], [24], [25]. The use of genetically engineered mouse models (GEMM) of breast cancer have allowed the isolation of residual and disseminated tumor cells in orthotopic, immunocompetent models [26]. Interestingly, in the GEMMs as in cancer patients, disseminated tumor cells can leave the primary tumor early during progression and remain dormant in distant sites before giving rise to metastases [11].

The MMTV-PyMT genetically engineered mouse has been shown to be a reliable model of metastatic breast cancer at the histologic and molecular levels [27]. The mouse mammary tumor virus (MMTV) promoter drives the expression of Polyoma Middle T-Antigen (PyMT) in the mammary epithelium and other organs [28]. PyMT is a membrane scaffold protein that activates the Ras/Raf/MEK and PI3K/Akt pathways [29]. These mice develop well-differentiated, luminal-type adenomas that progress to metastatic, poorly-differentiated adenocarcinoma [30], [31]. However, by adulthood the mice develop many thousands of tumor foci in their mammary glands, making it difficult to study progression of individual tumor foci. We recently described a hyperplasia transplant approach that can be used to track the progression of single MMTV-PyMT tumor foci from hyperplasia to adenoma to adenocarcinoma in an orthotopic, immunocompetent setting. Tumor cells are labeled with green fluorescent protein (GFP), allowing the isolation of residual tumor cells and disseminated tumor cells in distant sites [31]. Removal of the primary tumor models allows for metastatic outgrowth of disseminated cells. We have shown that tumor dissemination begins early during tumor progression at the early adenocarcinoma stage (8 weeks after transplantation), but metastases arise from more advanced adenocarcinomas (15 weeks after transplantation). Here we describe the characterization of residual tumor cells, disseminated tumor cells, and metastases from the MMTV-PyMT hyperplasia transplant model. The data identify novel biomarkers for the detection and targeting of these disease states and also provides a mouse model that can be used for future biomarker and efficacy studies.

## Materials and Methods

### Mouse Breeding and Tumor Transplantation

All mouse experiments were reviewed and approved by the Genentech Institutional Care and Use Committee (IACUC). MMTV-PyMT mice (FVB/N-Tg(MMTVPyVT)634Mul/J) and actin-GFP mice (FVB.Cg-Tg(CAG-EGFP)B5Nagy/J) were purchased from Jackson laboratory and relevant licenses were purchased for these experiments. MMTV-PyMT and actin-GFP mice were bred, and 3-week-old female bitransgenic offspring were sacrificed for collection of tumors, as described [31]. Focal, 0.5–1 mm hyperplastic tumors were microdissected with No. 15 scalpels (under guidance of an MZ10F fluorescent dissecting microscope) and placed in HBSS solution. Tumors were then implanted into cleared No. 4 mammary fat pads of 3 week-old female FVB/n mice (Charles River). Tumor measurements were made using electronic digital calipers and tumor volumes were calculated using the tumor ellipsoid formula (V = π/6×length×width^2^).

### Residual Tumor Cell Isolation

MMTV-PyMT transplant mice were treated with TAC chemotherapy every 21 days beginning at 8 weeks post-transplant. TAC chemotherapy regimen consisted of doxorubicin 5 mg/kg IV (Sigma), docetaxel 25 mg/kg IV (LC Laboratories), and cyclophosphamide 120 mg/kg IP (Sigma). Residual tumors (10 days after 1^st^ dose), relapsed tumors (10 days after 4^th^ dose), and untreated tumors were harvested, digested in 1 mg/ml collagenase/dispase (Roche) in DMEM-10%FBS for 2 hours, and single cell suspensions were prepared. GFP-positive tumor cells (300,000 cells) were collected by cell sorting (FACS Aria) and mRNA was immediately harvested (Qiagen RNeasy Micro kit) for microarray studies.

### Isolation of Disseminated Tumor Cells and Metastases

Lungs of mice bearing 18-week tumor outgrowths were harvested for disseminated tumor cells. To obtain metastases, primary tumors from mice bearing 18-week outgrowths were surgically removed, as described [31]. After primary tumor removal, mice were sacrificed at the first signs of respiratory distress (typically 8–10 weeks after surgery) to collect metastases. Lungs bearing disseminated cells or metastases were digested in 1 mg/ml collagenase/dispase (Roche) in DMEM-10% FBS for 2 hours, and single cell suspensions were prepared. GFP-positive tumor cells (300,000 cells) were collected by cell sorting (FACS Aria) and mRNA was immediately harvested (Qiagen RNeasy Micro kit) for microarray studies. The lungs of 3–6 18-week outgrowth mice were pooled to obtain sufficient numbers of sorted disseminated tumor cells for microarray profiling.

### Microarray Gene Expression Profiling

Quantity and quality of total RNA samples was determined using ND-1000 spectrophotometer (Thermo Scientific, Wilmington, DE) and Bioanalyzer 2100 (Agilent Technologies, Santa Clara, CA), respectively. For residual, relapsed, and untreated samples, total RNA was converted to double-stranded cDNA and then into Cy-dye labeled cRNA using Quick Amp Labeling Kit (Agilent). For adenoma, carcinoma, disseminated tumor, and metastasis samples, mRNA was amplified in two rounds and labeled using Message Amp II aRNA amplification kit (Applied Biosystems) and Quick Amp Labeling Kit (Agilent). For all samples, 750 ng of the labeled cRNA was fragmented and hybridized to the Agilent’s Whole Mouse Genome 4×44 Kv2 arrays, as described in manufacturer’s hybridization kit. Biological samples (n = 5 per group) were labeled with Cy5-dye and hybridized against Cy3-dye labeled Universal mouse reference (Stratagene, La Jolla, CA). Following hybridization, the arrays were washed, dried and scanned on Agilent’s microarray scanner. Agilent’s Feature Extraction software 10.7 was used to analyze acquired array images. Differential gene expression was analyzed with linear models for microarray data (Limma) and a normal exponential convolution model was applied for the background correction [32]. Loess method was applied for within-array normalization and a quantile method was applied for between-array normalization. False discovery rate (FDR) was estimated by Benjamini-Hochberg procedure. Microarray data was archived in the GEO Microarray Omnibus with the accession number GSE43566.

### Immunohistochemistry

Samples for histology were fixed overnight in 4% paraformaldehyde/PBS and paraffin embedded. Sections (5 µm) were cut, rehydrated, processed with citrate buffer antigen retrieval, and blocked with 5% BSA or serum (from secondary antibody host). Primary antibodies were diluted in 0.5× blocking solution and incubated on slides overnight at 4°C. Primary antibodies used include anti-Stat1 (Cell Signaling), anti-p-Stat1 (Cell Signaling), anti-F480 (AbD Serotec), and anti-phospho-H2A.X (Cell Signaling). For immunofluorescence, sections were incubated with biotinylated anti-rabbit (or goat) secondary antibody and then streptavidin-Alexa 488 or Alexa 564 (Molecular Probes). For immunohistochemistry, sections were incubated with biotinylated anti-rabbit (or goat) secondary antibody, streptavidin-HRP and then ABC signal amplification reagent, followed by DAB chromogenic detection (Vector Biolabs).

### RT-PCR

mRNA quality was assessed with Agilent Bioanalyzer and quantified with the Nanodrop instrument. 0.25–1 µg mRNA was reverse-transcribed to cDNA (Clontech Advantage kit) for 48×48 Fluidigm analysis, which was performed according to manufacturer’s instructions. Gene primers for RT-PCR were designed and validated by Fluidigm. All cDNA measurements were normalized to Gapdh expression. Independent sets of samples were used for RT-PCR, microarray profiling, and protein analysis.

### Western Blot

Tumors were harvested in RIPA buffer containing protease inhibitor/HALT phosphatase inhibitor cocktails (Thermo Scientific) and then homogenized (Powergen Tissue Homogenizer). Blotting for proteins was performed with the Licor Odyssey instrument. Antibodies for western blotting included anti-H3K4-tri-methyl (Abcam and ActiveMotif), anti-H3K4-di-methyl (ActiveMotif), anti-H3K4-dimethyl (ActiveMotif), anti-H3K27-tri-methyl (Millipore), anti-H3K9-tri-methyl (Abcam), anti-Histone-3 (ActiveMotif), anti-Stat1 (Cell Signaling), anti-p-Stat1 (Cell Signaling), anti-p-Stat3 (Cell Signaling), anti-p-Stat6 (Cell Signaling), and anti-β-actin (Abcam). In some cases (e.g., anti-H3K4-tri-methyl), antibodies from multiple sources were used to confirm blotting results.

## Results

### Profiling of Residual Tumor Cells in Chemotherapy-treated MMTV-PyMT Mice

**Figure 1:**
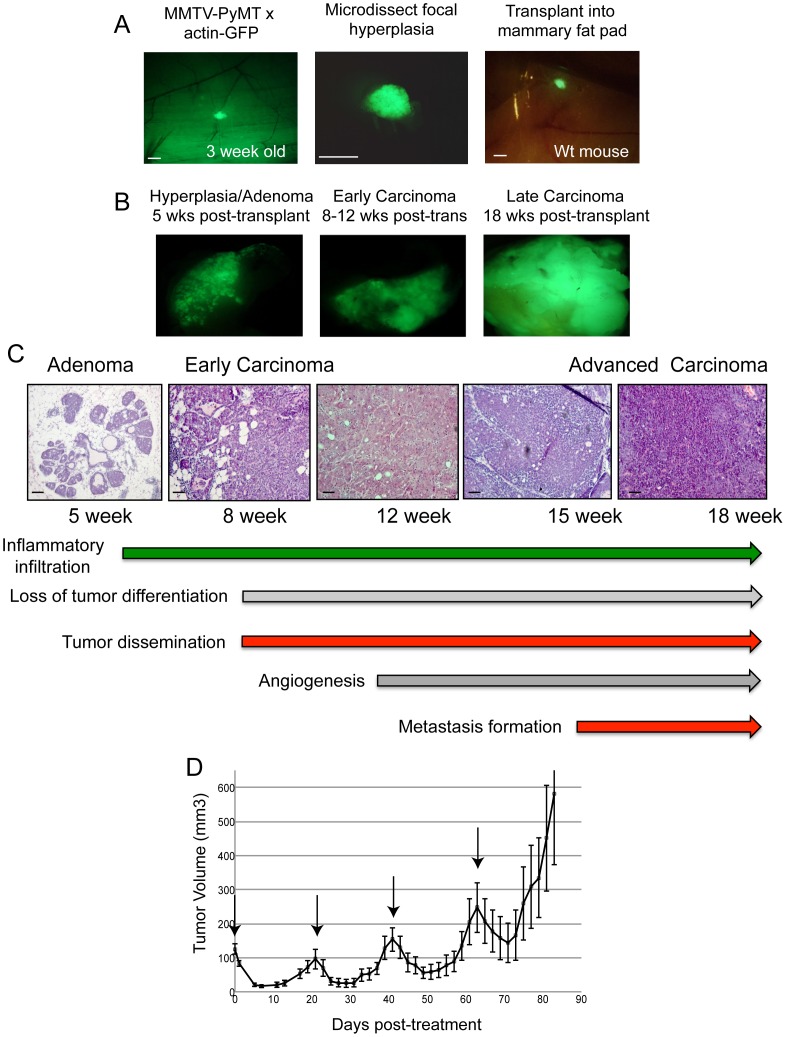
Modeling breast cancer residual disease in the MMTV-PyMT hyperplasia transplant model. (A) Representative images of a focal hyperplastic tumor in the No. 4 mammary gland of a female, 3 week-old MMTV-PyMT;actin-GFP mouse (left panel). The tumor was microdissected from mammary gland, placed in PBS (middle image), and then transplanted into the mammary fat pad of a wild-type mouse (right panel). (B) and (C) Representative fluorescent and H&E images of tumor outgrowths after transplantation. (D) Tumor response curve of MMTV-PyMT transplant mice treated with TAC chemotherapy (docetaxel, doxorubicin, cyclophosphamide); mean ± s.e.m., n = 10. Arrows indicate chemotherapy dosing. Scale bars correspond to 1 mm (A) and 100 um (C).

To determine the response of single tumor foci to cytotoxic chemotherapy, we utilized the MMTV-PyMT hyperplasia transplant system [31]. Focal hyperplasias were resected from 3-week-old MMTV-PyMT;actin-GFP mice and orthotopically transplanted into the cleared mammary fat pads of FVB/n host mice (Figure 1A). Tumor outgrowths displayed stereotyped tumor growth and histologic progression as compared to MMTV-PyMT mice, with progression to early carcinoma and late carcinoma occurring at 8 and 15 weeks post-transplantation, respectively (Figure 1B). The hyperplasia transplant model is amenable for studying the individual steps in malignant progression, such as angiogenesis, tumor dissemination, and metastasis formation (Figure 1C).

Tumor-bearing mice at the early carcinoma stage (8 weeks post-transplant) were treated with cytotoxic chemotherapy to identify residual tumor cells. We modeled the clinical setting by administering the TAC chemotherapy regimen (taxol, adriamycin, cyclophosphamide), which is given to patients with poorly-differentiated breast adenocarcinoma [33]. Tumor-bearing mice were treated with 25 mg/kg docetaxel, 5 mg/kg doxorubicin, and 120 mg/kg cyclophosphamide every 21 days, similar to patients. Mice lost 8–10% body weight after each dose but recovered within 21 days of dosage (data not shown). Tumors underwent significant regression within seven days after the first dose of chemotherapy. By day 10, tumors stabilized and started to grow once again (Figure 1D). Within 21 days after the first dose, the tumors relapsed and grew nearly to the size of the original tumor. Subsequent administration of chemotherapy every 21 days led to similar patterns of tumor regression and relapse (Figure 1D). We collected the untreated tumors, residual tumors at day 10, and relapsed tumors at day 80 for further analysis. GFP-positive tumor cells from these samples were FACS-sorted, and RNA was harvested for mRNA microarray profiling.

### Enrichment of Jak/Stat, Notch, and Epigenetics Genes in Residual Tumors

**Figure 2:**
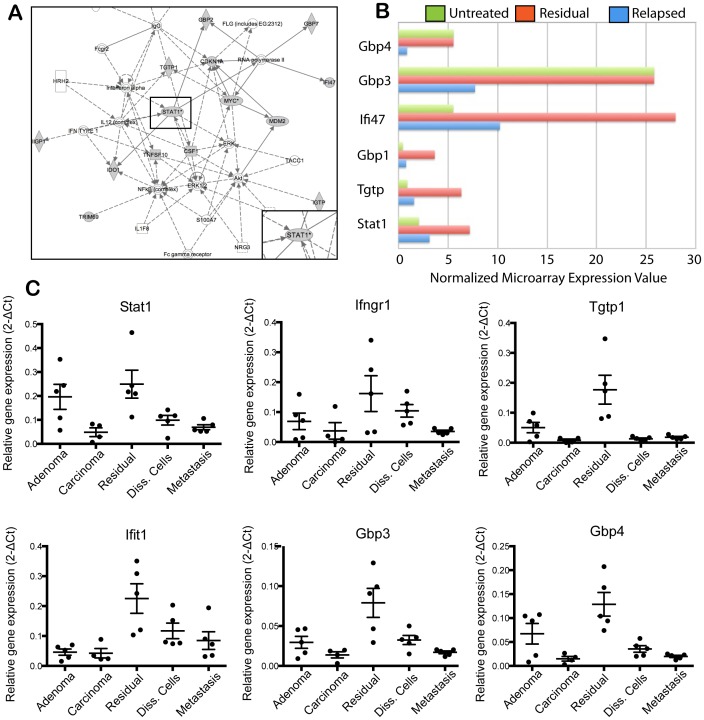
Ifn-γ/Jak/Stat signaling in MMTV-PyMT residual tumor cells after chemotherapy. (A) Ingenuity pathway analysis of genes up-regulated in chemotherapy-treated residual tumors in the MMTV-PyMT transplant model. (B) Microarray expression values of Ifn-γ/Jak/Stat-associated genes in untreated (green), residual (red) and relapsed (blue) tumors; mean, n = 5 per group. (C) Fluidigm RT-PCR analysis of Ifn-γ/Jak/Stat-associated genes. GFP-positive tumor populations were FACS-sorted from the indicated groups and mRNA was harvested for the analysis; mean ± s.e.m., n = 5 per group. Data was normalized to Gapdh expression.

Microarray profiling of untreated, residual, and relapsed tumors revealed a number of signaling pathways and biomarkers enriched in residual tumors (GEO Microarray Omnibus, accession number GSE43566). These include the Jak/Stat pathway, DNA damage response/repair pathways (Atm/Atr and Brca1-mediated), and Akt signaling pathways (Table 1). Ingenuity pathway analysis of microarray data identified Stat1 as an important signaling node in residual tumors (Figure 2A). Interestingly, several genes in the Ifn-γ/Jak/Stat pathway, such as Gbp1, Gbp3, Gbp4, Ifi47, Tgtp, and Stat1, were up-regulated in residual tumors relative to untreated and/or relapsed tumors (Figure 2B). We validated the enrichment of these genes in residual tumors with Fluidigm RT-PCR. GFP-positive tumor cells were sorted from adenomas (5-week outgrowths), carcinomas (18-week outgrowths), residual tumors, disseminated tumor cells in lungs, and lung metastases for this analysis. In agreement with microarray data, several members of the Ifn-γ/Jak/Stat pathway, including Stat1, Ifngr1, Tgtp1, Ifit1, Gbp3, and Gbp4, were significantly up-regulated in residual tumors relative to the other groups (Figure 2C). A subset of the genes, such as Stat1 and Gbp4, were also up-regulated in adenoma tumors, suggesting that elements of the pathway were active in early breast tumors.

**Table 1:** Gene families enriched in residual MMTV-PyMT tumors after chemotherapy.

Gene Family	p-value	Gene Name
Interferon signalingDNA damage_Brca1-mediated	0.0000030.00003	STAT1, GBP1, GBP4, IFI47, TGTP, IRF1, IGTPSTAT1, CDKN1A, Myc
DNA damage_ATM/ATR-mediated	0.00004	MDM2, CDKN1A, Myc
Cytoskeleton remodeling	0.00007	Wnt6, Myc
Akt signaling pathway	0.00009	
IFN-gamma signaling pathway	0.0002	STAT1, GBP1, GBP4, IFI47, TGTP, IRF1, IGTP
Thrombopoetin signaling via JAK/STAT	0.0009	
IL-27 signaling pathwayETV3 signaling pathway	0.0010.002	
PDGF signaling via STATs and NF-kB	0.002	

**Figure 3:**
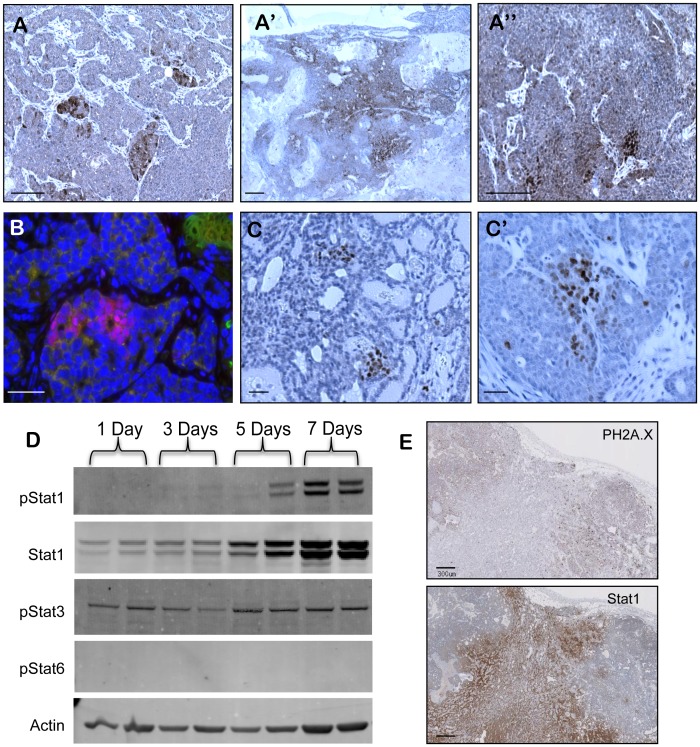
Enrichment of a Stat1-positive tumor sub-population in MMTV-PyMT residual tumor cells after chemotherapy. (A–A″) Immunohistochemistry of Stat1 in untreated tumors (A), residual tumors (A’) and relapsed tumors (A″). (B) Immunofluorescence of Stat1 (red), F4-80 (green), and DAPI (blue) in untreated tumors sample. (C–C′) Immunohistochemistry of p-Stat1 in untreated (C) and residual tumor samples (C′). (D) Western blot of p-Stat1, Stat1, p-Stat3, p-Stat6, and actin control in MMTV-PyMT whole tumor lysates. Mice were treated with TAC chemotherapy and tumors harvested on the indicated number of days after treatment. (E) Immunohistochemistry of Stat1 and phospho-H2A.X in residual tumor sample. Scale bars correspond to 200 um (A–A″), 300 um (G) and 25 um (B–E′).

We further validated the enrichment of Jak/Stat pathway genes in residual tumors at the protein level. Stat1 immunohistochemistry of untreated MMTV-PyMT adenocarcinomas identified sub-populations of Stat1-positive tumor cells (Figure 3A). The number of Stat1 positive cells and the ratio of Stat1 positive-to-negative cells increased significantly in residual tumors after chemotherapy (Figure 3A′). Relapsed tumors contained only small Stat1 sub-populations, much like untreated tumors, suggesting that enrichment of Jak/Stat genes was specific to the residual disease state (Figure 3A″). Co-immunofluorescence with a macrophage marker showed that Stat1 was expressed in tumor cells, as was reported in breast cancers (Figure 3B) [34]. Further, p-Stat1-positive cells were detected in untreated and residual tumors. Interestingly, p-Stat1-positive tumors cells were also found in clustered sub-populations in tumors (Figure 3C–C′).

We determined the kinetics and mechanism of Stat1 activity in residual tumors after chemotherapy. We collected tumor lysates at days 1, 3, 5, and 7 after chemotherapy and blotted for Stat1 and p-Stat1, -3, and -5 proteins (Figure 3D). Stat1 and p-Stat1 levels were significantly increased by day 7 after chemotherapy, corresponding to development of residual disease. Interestingly, Stat1-positive tumor cells in residual tumors displayed significantly less DNA damage than Stat1-negative tumor cells (Figure 3E). This suggested that Stat1 positive tumor cells, which pre-existed in untreated tumors, were inherently resistant to DNA-damaging agents, as reported [35], [36]. This may result from more active DNA repair mechanisms in Stat1 positive cells [37]. The increased DNA repair activity of Stat1-positive tumor cells may have primed tumor cells for drug tolerance and tumor relapse after chemotherapy in this model.

**Figure 4:**
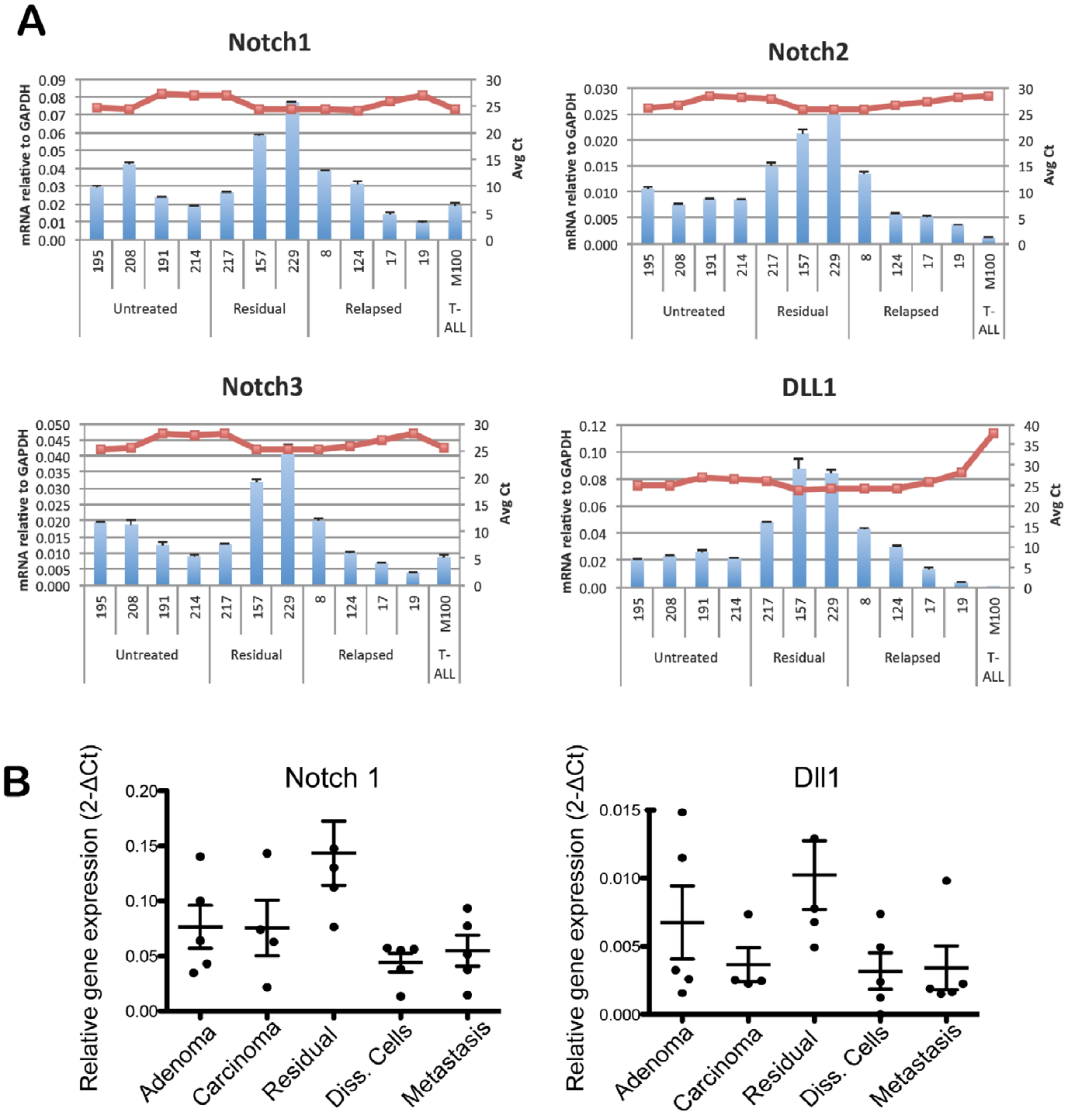
Up-regulation of Notch family members in MMTV-PyMT residual tumors. (A) Taqman RT-PCR analysis of untreated, residual and relapsed tumors. Whole tumor lysates were harvested and mRNA purified for RT-PCR. Individual tumor samples were analyzed in triplicate; mean ± s.e.m, red line indicates average Ct value. M100 sample was negative control. (B) Fluidigm RT-PCR analysis of Notch1 and Dll1 in tumor populations. GFP-positive tumor populations were FACS-sorted from indicated groups and mRNA was harvested for the analysis; mean ± s.e.m., n = 5 per group. All data were normalized to Gapdh expression.

In addition to Jak/Stat family genes, we identified Notch family members as biomarkers of residual tumor disease in this model. We first determined the expression levels of Notch family members in whole tumor lysates, which included both tumor and stromal populations. Taqman RT-PCR analysis confirmed the up-regulation of Notch-1, Notch-2, Notch-3, and Dll-1 in residual tumors relative to untreated or relapsed tumors (Figure 4A). We then performed Fluidigm RT-PCR analysis on sorted tumor populations to determine the tumor cell specificity of Notch gene expression. Sorted residual tumor cells were compared to sorted tumor cells from adenomas, carcinomas, disseminated tumor cells, and lung metastases. We found that Notch-1 and Dll1 were specifically up-regulated in residual tumors compared to primary tumors, disseminated cells, or metastases. Dll1 demonstrated elevated expression in both residual and untreated adenoma tumors, similar to Stat1 expression (Figure 4B).

**Figure 5:**
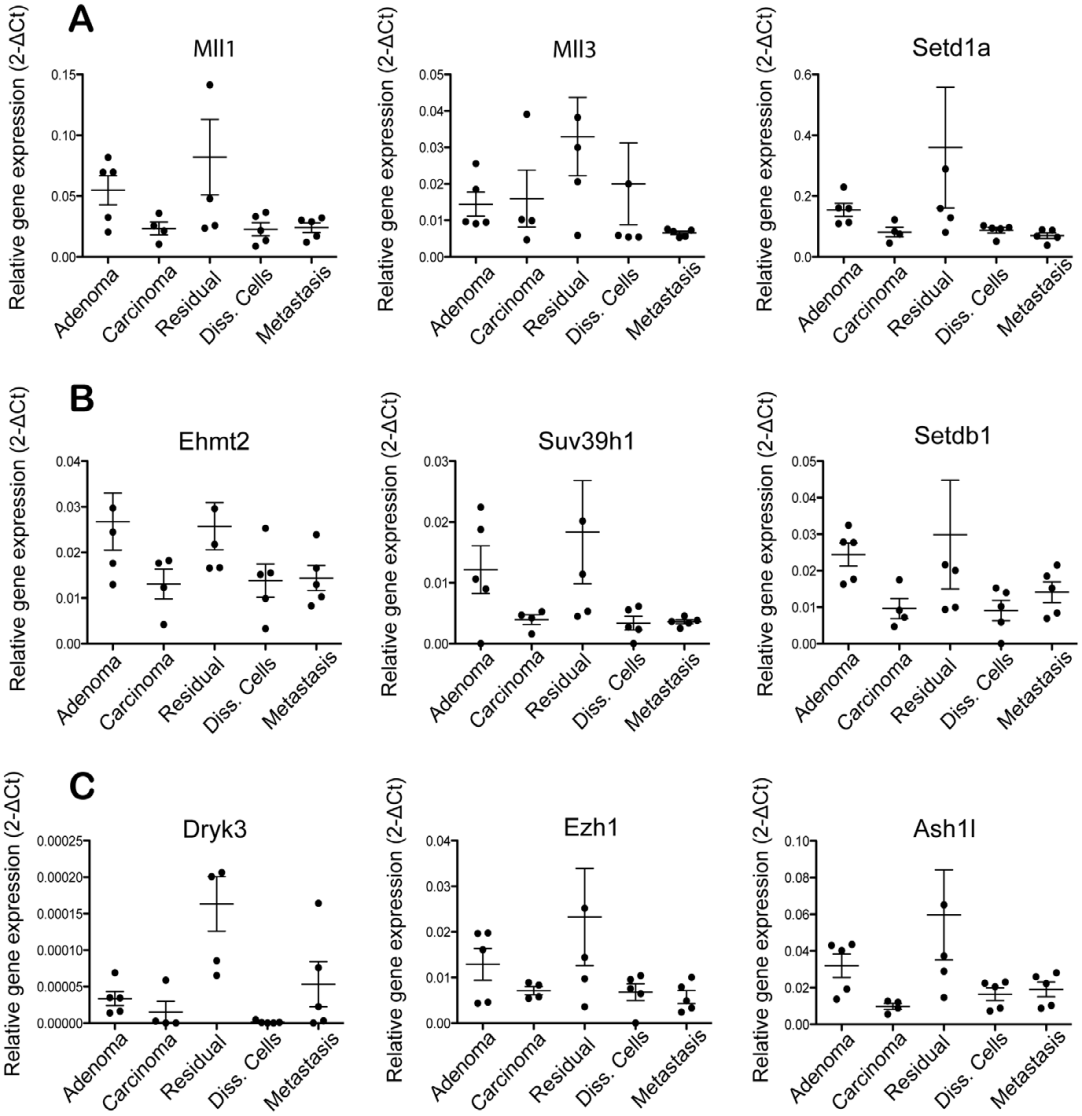
Up-regulation of chromatin modifying gene in MMTV-PyMT residual tumors. Fluidigm RT-PCR analysis of H3K4 methyltransferases (A), H3K9 methyltransferases (B) and other chromatin-modifying enzymes (C) in various tumor populations. GFP-positive tumor populations were FACS-sorted from indicated groups and mRNA was harvested for the analysis; mean ± s.e.m., n = 5 per group. Data were normalized to Gapdh expression.

A number of chromatin-modifying genes, in particular histone methyltransferases, were significantly up-regulated in residual tumors. We validated the enrichment of these genes in sorted residual tumor cells by Fluidigm RT-PCR analysis. A number of methyltransferases specific to H3K4 (Mll1, Mll3, Setd1a), H3K9 (Ehmt2, Suv39h1, Setdb1) and H3K27 (Ezh1) were up-regulated in residual tumors compared to untreated primary tumors, disseminated cells, or metastases (Figure 5A–C). Dyrk3 (a histone-modifying kinase) and Ash1l (a methyltransferase) were also enriched in residual tumors (Figure 5C). In some cases, these genes were uniquely up-regulated in residual tumors (e.g., Setd1a, Dyrk3), while in other cases the genes were up-regulated in both residual and untreated adenoma tumors (e.g., Suv39h1, Mll1) (Figure 5A–C).

### Profiling of Disseminated Tumor Cells and Metastases in MMTV-PyMT Mice

**Figure 6:**
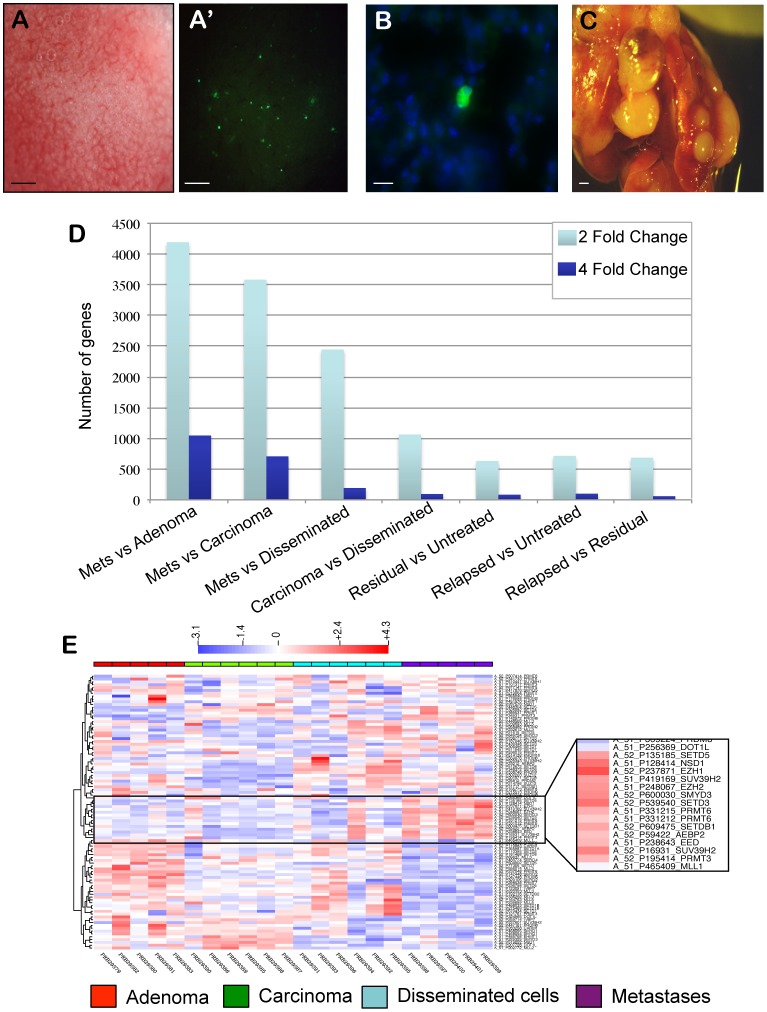
Profiling of disseminated cells and metastases in MMTV-PyMT hyperplasia transplant model. Representative brightfield (A) and GFP fluorescence (A′) images of the lungs of mice with advanced carcinoma (18 week outgrowths). Disseminated tumors cells were detected as single, GFP-positive cells in the lungs as early as 8-weeks post-transplant. (B) Representative image of a single disseminated tumor cell in the lung of an 18-week-tumor-bearing mouse. Green indicates GFP and blue indicates DAPI. (C) Whole mount image of lung metastases. Mice with 18-week tumor outgrowths were anesthetized and the primary tumor was surgically removed. Mice were sacrificed when they developed the first signs of respiratory distress, indicating the growth of lung metastases (typically 8–10 weeks after primary tumor removal). (D) Number of genes differentially expressed by 2-fold (light blue) and 4-fold (dark blue) in microarray dataset comparisons. (E) Hierarchical clustering of chromatin modifying genes in tumor populations. Tumor populations from adenoma (red), carcinoma (green), disseminated cells (blue), and metastases (purple) were FACS-sorted for profiling. Inset shows clustering of H3K4, H3K9, and H3K27 methyltransferases. Scale bars correspond to 1 mm (A, C) and 5 um (B).

The MMTV-PyMT hyperplasia transplant model allows the collection of disseminated tumor cells in various organs, including lung, spleen, brain, and liver (Figure 6A–A′). The vast majority of disseminated cells are single cells in the microvasculature of distant organs (Figure 6B) [31]. The number of disseminated tumor cells in distant organs rises in proportion to tumor size. In 18-week adenocarcinoma outgrowths, >5000 disseminated tumor cells can be detected in the lungs of tumor-bearing mice. However, primary tumor removal experiments showed that less than 0.1 percent of these disseminated tumor cells in lung are capable of forming lung metastases. Further, although disseminated cells can be detected in many organs, metastases only arise in the lungs (Figure 6C) [31]. This model can be used to determine the mechanisms of tumor dissemination and metastasis formation in an orthotopic, immunocompetent setting.

We FACS-sorted GFP-positive tumor cells from adenomas (5 week outgrowths), carcinomas (18 week outgrowths), disseminated tumor cells from lungs, and lung metastases for microarray expression profiling (GEO Microarray Omnibus, accession number GSE43566). When compared to primary tumors or disseminated cells, lung metastases showed substantial differences in gene expression, with 3500–4000 genes differentially expressed (2-fold level) relative to adenoma or carcinoma (Figure 6D). For comparison, chemotherapy-treated residual or relapsed tumors had ∼600 differentially expressed genes (2-fold level) relative to untreated tumors. Interestingly, disseminated cells appeared more similar to carcinoma than to metastases (1000 versus 2400 differentially expressed genes, respectively) (Figure 6D). These data indicate that lung metastases in the MMTV-PyMT model are molecularly distinct at the mRNA expression level from primary tumors or disseminated tumor cells.

### Enrichment of JAK/STAT Genes and Epigenetic Regulators in Disseminated Tumor Cells and Metastases

We performed hierarchical clustering and pathway analysis of genes differentially expressed in metastases relative to primary tumors. The gene families most represented in differentially expressed genes were stem-cell-associated markers, including markers of neural and embryonic stem cells (Figure S1). A number of stem-cell-related genes, such as Fzd3, Xiap, Sox2, and several Smad members were up-regulated in metastases relative to primary tumors. Given the substantial up-regulation of stem cell-associated genes, including cell fate determination genes and transcription factors, in metastases relative to primary tumors, we hypothesized that metastases had undergone epigenetic reprogramming during cancer progression. To test this, we performed hierarchical clustering of chromatin-modifying gene expression in metastases, disseminated tumor cells and primary tumors. We identified a cluster of lysine and arginine methyltransferases that were highly expressed in metastases (Figure 6E). Quantitative analysis of microarray data confirmed that these methyltransferases, which included Setd3, Setd5, Suv39h2, Smyd3, Prmt3, Prmt6, Nsd1, and Nsd2 were up-regulated in metastases compared to adenomas, carcinomas, or disseminated cells (Table S1). A number of these genes catalyze the methylation of H3K4 and H3K9 residues. The enrichment of these methyltransferases suggested that metastases had acquired specific epigenetic markers during cancer progression.

**Figure 7:**
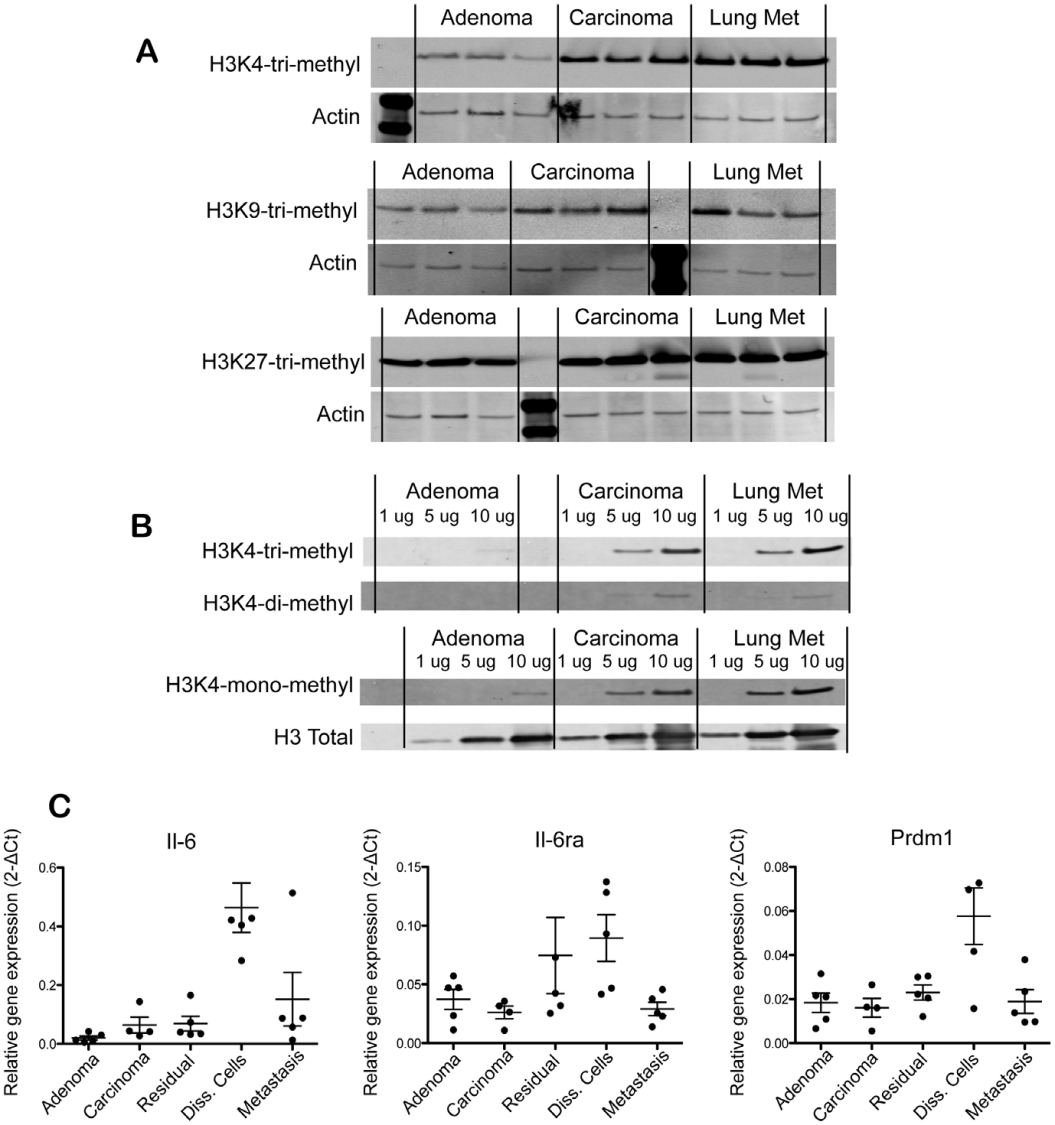
Histone-3 methylation marks in cancer progression. (A) Western blot of H3K4 tri-methyl, H3K9 tri-methyl, H3K27 tri-methyl and actin control in tumor lysates from adenomas (5 week outgrowth), carcinomas (18 week outgrowth), and lung metastasis; n = 3 per group. (B) Western blot of H3K4 tri-methyl, H3K4 di-methyl, H3K4 mono-methyl and Histone 3 control performed with serial dilutions of tumor lysates (1 ug, 5 ug, 10 ug total protein loaded per well). (C) Fluidigm RT-PCR analysis of Il-6, Il-6ra, and Prdm1 in the indicated tumor populations. GFP-positive tumor populations were FACS-sorted from the indicated groups and mRNA was harvested for the analysis; mean ± s.e.m., n = 5 per group. Data was normalized to Gapdh expression.

We assessed the methylation status of H3K4, H3K9, and H3K27 in metastases and primary tumors in the MMTV-PyMT transplant model. Lung metastases had higher H3K4 trimethylation relative to adenomas and carcinomas, although the majority of this increase could be accounted for in the adenoma-carcinoma transition (Figure 7A). H3K9 trimethylation was more heterogeneous across tumors and metastases. Metastases demonstrated significantly elevated H3K9 trimethylation relative to tumors. In contrast, metastases had similar or slightly elevated levels of H3K27 trimethylation relative to adenomas or carcinomas (Figure 7A). Further analysis of H3K4 methylation status revealed that H3K4 tri-methyl, H3K4 di-methyl and H3K4 mono-methyl marks were increased in metastases relative to primary tumors. In all cases, carcinomas had increased levels of these markers relative to adenomas. Interestingly, H3K4 tri-methyl and H3K4 mono-methyl marks appeared more represented than H3K4 di-methyl across the tumor types (Figure 7B).

Mining of microarray data was performed to identify biomarkers of disseminated tumor cells in this model. The Il-6/Jak/Stat pathway members emerged as biomarkers for disseminated tumors cells in lungs (Figure 7C). Il-6 mRNA levels were significantly up-regulated in disseminated cells relative to metastases, primary tumors or residual tumors. Il6ra was also enriched in disseminated cells, though it was also elevated in in residual tumors. Prdm1, a transcriptional repressor and effector of Il-6 signaling, was also uniquely up-regulated in disseminated tumor cells, suggesting that active Il-6/Jak/Stat signaling occurs in this population (Figure 7C).

## Discussion

### Jak/Stat Pathway in Residual Tumors and Disseminated Tumor Cells

We have used the MMTV-PyMT hyperplasia transplant model to identify biomarkers of disseminated tumor cells and residual tumors persisting after chemotherapy. These cell populations are often undetectable in cancer patients until they give rise to recurrent primary tumors or distant metastases. Identifying these rare cell populations may allow the early detection and treatment of these disease states in cancer patients. GEMMs and other models of cancer may aid in identifying biomarkers of these rare disease states. Biomarker identification may also lead to a better understanding of the biology underlying residual disease, tumor dissemination, and metastasis formation. In the MMTV-PyMT model, the tumor outgrowths initially resemble luminal-type breast cancer, but with malignant progression the tumors develop into basal-like breast cancer. Thus, distinct human breast cancer subtypes can be represented in this model depending on experimental setting.

Jak/Stat pathway genes emerged as biomarkers of residual disease and disseminated tumor cells in the MMTV-PyMT model. Human breast cancer samples have been known to contain high levels of p-Stats, in particular p-Stat1, p-Stat3, and p-Stat5 [38], [39]. Stat1 and Stat3 are expressed in both the tumor cell and stromal cell compartments of breast cancers [34]. Recent microarray studies have shown high expression levels of Stat1 and Stat3 in primary breast cancers, with several studies grouping Stat1 within the top one percent of most highly expressed genes [40], [41], [42]. Stat1 showed significant variation of expression across breast cancer samples [40]. Microarray analysis of Ifn-γ/Jak/Stat1 effector genes, such as Gbp1 and Gbp5, have grouped the pathway with estrogen receptor negative (ER-) and triple-negative (ER−/PR/Her2-) breast cancers [1], [43], [44], [45], [46]. A recent study demonstrated activation of the IL-6/Jak/Stat pathway in basal-like breast cancer cells *in vitro* and *in vivo*. An IL-6/Jak/Stat gene signature predicted higher rates of metastasis formation in breast cancer patients [47]. These studies suggest that Jak/Stat pathway members are highly expressed in a subset of breast cancers with poor prognosis.

The role of Jak/Stat pathway in residual disease may be linked to its described roles in drug tolerance/resistance, cell survival pathways, and DNA repair mechanisms. A number of studies have reported up-regulation of the Ifn-γ/Jak/Stat1 pathway in cancer cell lines and xenograft models that developed resistance to chemotherapeutic agents and/or irradiation. These studies span a number of cancer types, including breast, prostate, lung, ovary, myeloma, and melanoma [35], [36], [48], [49], [50], [51], [52], [53], [54]. The mechanisms of Stat1-mediated resistance may in part be due to activation of ATM-dependent DNA checkpoint pathways, p-glycoprotein, and cell survival pathways [37], [55]. Other studies reported up-regulation of the Il-6/Jak/Stat3 pathway in acquired resistance to chemotherapeutic agents or radiation [49], [56], [57], [58]. In one study, doxorubicin treatment of an *in vivo* lymphoma model led to the release of Il-6 in the tumor microenvironment, creating a chemoprotective niche for maintenance of residual cells [56]. In many studies, the Jak/Stat pathway causally regulated the development of drug resistance, such that Stat1 knockdown or JAK inhibitors led to chemosensitization of resistant tumors [35], [36], [49], [50], [56]. In breast cancer patients, an interferon-related gene signature, which included Stat1 and interferon-γ-induced genes, is predictive of poor response to chemotherapy or radiation [50]. These data suggest that in both the clinical and *in vitro* settings, Ifn-γ/Jak/Stat1-positive breast cancers are resistant to cytotoxic agents. Our data suggest that several pathway members, such as Stat1, Ifngr2, Tgtp1, Ifit1, Gbp1 and Gbp3, may be suitable biomarkers for residual disease in breast cancer patients treated with cytotoxic chemotherapy.

We also identified Il-6/Jak/Stat3 pathway genes as biomarkers of disseminated tumors cells in the MMTV-PyMT model. Il-6, Il-6ra and Prdm1 were specifically up-regulated in disseminated tumors cells but not in carcinomas, suggesting *de novo* expression of these genes in the microenvironment of the distant organ. In breast cancer patients, serum Il-6 levels correlate with the presence of metastasis and disease prognosis [59], [60]. As in residual cells, the role of Il-6/Stat3 in tumor dissemination may be linked to activation of cell growth and survival pathways [61], [62]. Il-6 is also known to have pro-angiogenic and pro-tumorigenic inflammatory properties in many cancer types [63]. For these reasons, Il-6 has been evaluated as a therapeutic target for several cancers, and early studies indicate that blocking Il-6 may lead to disease stabilization due to reduced tumor inflammatory infiltration and tumor angiogenesis [64]. Selective Jak1 and Jak2 inhibitors can also inhibit Il-6/Stat3 signaling and concomitantly reduce tumor growth in xenograft models [65]. These data suggest that Il-6 and Il-6-associated genes may serve as biomarkers for disseminated tumor cells and that Il-6-antagonists or Jak inhibitors may target this disease state in cancer patients.

### Notch Pathway in Residual Tumors

A number of Notch family members emerged as biomarkers of residual tumors in the MMTV-PyMT transplant model. Expression profiling indicated that Notch-1, Notch-2, Notch-3, and Dll-1 were up-regulated in residual tumors compared to untreated or relapsed tumors. Interestingly, Notch-1 and Dll1 were specifically up-regulated in residual tumors compared to untreated primary tumors, disseminated cells, or metastases, suggesting a unique role for these genes in residual tumor biology. A number of studies have linked Notch family members to cancer stem cells and the drug resistance [66], [67]. Further work will be needed to define the roles of cancer stem cells in residual tumor biology in this model. Additionally, the emergence of cytokine and Notch pathways, which rely on epithelial-stromal crosstalk, suggests an important role for the tumor-associated stroma in residual tumor biology. Further work will be needed to define the roles of stromal-associated genes as biomarkers and drivers of tumor relapse.

### Epigenetic Regulators as Biomarkers of Residual Tumors and Metastases

Recent studies indicate that histone-modifying proteins, in particular histone methyltransferases, are important drivers of cancer incidence and progression. Histone modifications by phosphorylation, methylation, acetylation, or ubiquitylation are important post-translational regulators of gene expression and DNA damage responses. Histone methylation can occur on arginine, lysine, and histidine residues on histone tails, and the most extensively studied modifications lie on histone 3 (H3) and histone 4 (H4). These modifications include H3K4, H3K9, H3K27, H3K36, H3K79, and H4K20 [68]. Three families of histone methyltransferases catalyze the methylation of lysine and arginine residues on histone tails. The SET-domain-containing proteins (consisting of 48 genes in the human genome) and Dot1l families can methylate lysine residues on histone tails, while the Prmt family methylates arginine residues on histone tails. Two families of demethylases have been identified that catalyze the demethylation of methyl groups on histone lysine residues [68]. Mutations, amplifications, deletions, and rearrangements of these epigenetic modifiers have been linked to cancer incidence and prognosis [68], [69], [70]. For example, translocations involved the H3K36 methyltransferase Nsd1 are linked to leukemias, whereas activating point mutations in the H3K27 methyltransferase Ezh2 are linked to B cell lymphoma [69]. Further, specific histone lysine methylation states in cancers have been linked to poor outcome (e.g., H3K9 trimethylation in gastric adenocarcinoma) [68].

A number of epigenetic regulators, in particular histone lysine methyltransferases, emerged as biomarkers of residual tumors in the MMTV-PyMT model. We identified specific H3K4 and H3K9 methyltransferases, such as Mll, Mll3, Setd1a, Ehmt2, Suv39h1, and Setdb1 as biomarkers of residual tumor cells persisting after chemotherapy. Histone modifying proteins and histone variants are important drivers of DNA damage response, DNA break repair, and checkpoint arrest in chemotherapy-treated and irradiated cells [71]. It is possible that the histone methyltransferases may regulate the DNA damage responses that allow the emergence of drug tolerance or resistance in residual tumors. However, chromatin-modifying genes have been linked to other signaling pathways that could drive residual disease biology. For example, the emergence of drug-tolerant persisters in cancer cell lines treated with various anti-cancer agents involved alteration of H3K4 demethylation linked to the demethylase Kdm5a. The mechanism of H3K4-demethylation appeared to involve Igf1R activity [19]. Further studies will be necessary to define the precise role of these histone methyltransferases in residual disease biology in the MMTV-PyMT and other cancer models.

Histone-lysine modifications and histone methyltransferases also emerged as biomarkers of metastases in the MMTV-PyMT model. H3K4 tri-methyl, H3K4 di-methyl, H3K4 mono-methyl, and H3K9 tri-methyl marks were all increased in lung metastases compared to primary tumors, although H3K9 trimethylation was more heterogeneous across metastases relative to H3K4 marks. A number of histone methyltransferases, including Setd3, Setd5, Suv39h2, Smyd3, Prmt3, Prmt6, Nsd1, and Nsd2, were up-regulated in metastases compared to disseminated tumor cells or primary tumors. The specific up-regulation of these genes in metastases suggested that they might play a role in metastasis biology. Histone lysine methyltransferases, such as the H3K36 methyltransferase Whsc1/Nsd2 and the H3K9 methyltransferase G9a/Ehmt2, have been shown to play critical roles in tumor invasion and metastasis formation [72], [73]. Specific histone markers are also known to correlate with metastasis formation and poor prognosis in cancer patients, further implying an epigenetic regulation of metastasis formation [68]. Further studies will be required to assess the epigenetic role of histone methyltransferases in metastasis formation in the MMTV-PyMT model.

### Future Directions

This study identifies biomarkers of residual disease, disseminated tumors cells, and metastases in a widely used mouse model of breast cancer. Residual tumors and disseminated tumor cells in breast cancer patients are rare cell populations that are difficult to isolate and characterize. Disseminated tumors cells have been isolated from bone marrow of breast cancer patients but not from common sites of metastases, such as liver and lung [11]. Recently, circulating tumor cells have been isolated from cancer patients for molecular profiling [26]. The biomarkers identified in this study can be further tested for their ability to detect residual disease and tumor dissemination in cancer patients. The identification of biomarkers in other mouse models of cancer can also lead to hypothesis generation as to the biological mechanisms of residual disease, tumor relapse, and metastasis formation.

## Supporting Information

Figure S1 **Enrichment of stem-cell associated genes in metastases.** (A) Hierarchical clustering of stem-cell associated gene families in adenomas (orange) and lung metastases (red). (B) Microarray expression values of stem-cell-related genes in adenomas (dark blue), carcinoma (light blue), disseminated cells (yellow) and metastases (red). GFP-positive tumor cells were FACS sorted and mRNA harvested for microarray expression profiling; n = 5 per group, * indicates adjusted-p<0.01 between metastasis and adenoma (t-test).
(TIF)

Table S1 **Enrichment of epigenetic regulators in disseminated cells and metastases.** Microarray normalized gene expression values of epigenetic-related genes in adenoma (A), carcinoma (C), disseminated cells (D), and metastases (M) samples. Tumor cells were FACS sorted and mRNA harvested for microarray profiling. p-values indicated for T-tests performed between A/M and C/D groups (n = 5 per group).
(DOC)
