# Supplementary figures and images for: Biomarkers of Residual Disease, Disseminated Tumor Cells, and Metastases in the MMTV-PyMT Breast Cancer Model
Source: PLoS One. 2013 Mar 8;8(3):e58183. doi: 10.1371/journal.pone.0058183 (PMC3592916; doi:10.1371/journal.pone.0058183)

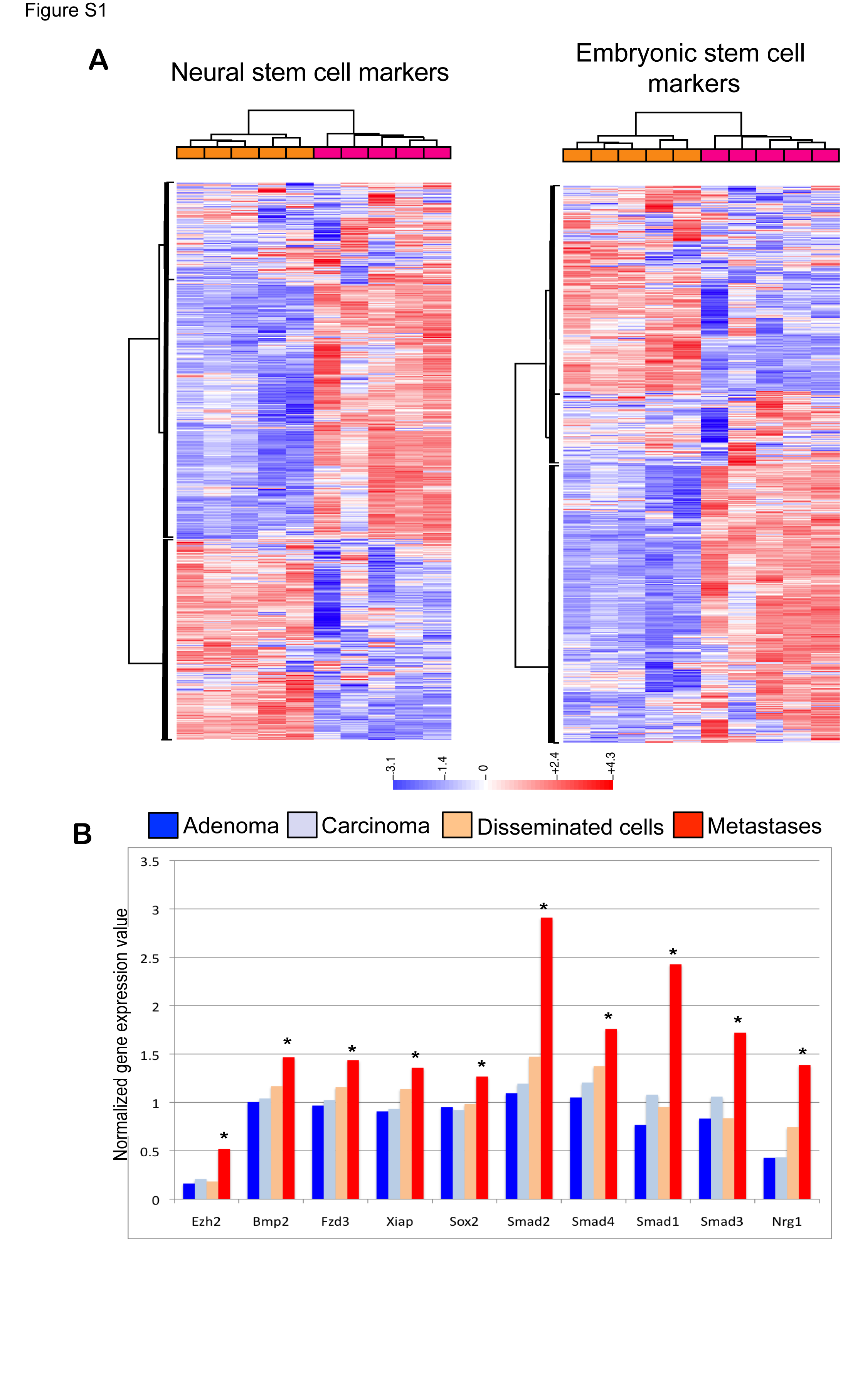

Supplement: Figure S1 — Enrichment of stem-cell associated genes in metastases. (A) Hierarchical clustering of stem-cell associated gene families in adenomas (orange) and lung metastases (red). (B) Microarray expression values of stem-cell-related genes in adenomas (dark blue), carcinoma (light blue), disseminated cells (yellow) and metastases (red). GFP-positive tumor cells were FACS sorted and mRNA harvested for microarray expression profiling; n = 5 per group, * indicates adjusted-p<0.01 between metastasis and adenoma (t-test). (TIF) [file pone.0058183.s001.tif]
