# Supplementary material for: Biomarkers of Residual Disease, Disseminated Tumor Cells, and Metastases in the MMTV-PyMT Breast Cancer Model
Source: PLoS One. 2013 Mar 8;8(3):e58183. doi: 10.1371/journal.pone.0058183 (PMC3592916; doi:10.1371/journal.pone.0058183)
Supplement: Table S1 — Enrichment of epigenetic regulators in disseminated cells and metastases. Microarray normalized gene expression values of epigenetic-related genes in adenoma (A), carcinoma (C), disseminated cells (D), and metastases (M) samples. Tumor cells were FACS sorted and mRNA harvested for microarray profiling. p-values indicated for T-tests performed between A/M and C/D groups (n = 5 per group). (DOC) [file pone.0058183.s002.doc]

| Table S1. Microarray normalized gene expression values of epigenetic regulators in adenoma (A), carcinoma (C), disseminated cells (D), and metastases (M) | | | | | | |
| --- | --- | --- | --- | --- | --- | --- |
| Gene Name | Adenoma | Carcinoma | Disseminated  Cells | Metastases | p-value  (M, A) | p-value  (C, D) |
| Mll1 | 2.16 | 1.20 | 1.40 | 0.74 | 0.0028 | 0.6718 |
| Mll3 | 0.78 | 0.52 | 0.64 | 0.16 | 0.0001 | 0.5656 |
| Mll5 | 0.30 | 0.35 | 0.34 | 0.16 | 0.0358 | 0.9397 |
| Setd1a | 1.58 | 0.80 | 1.21 | 0.78 | 0.0070 | 0.0823 |
| Setd1b | 1.79 | 1.09 | 2.44 | 0.71 | 0.0053 | 0.0095 |
| Setdb1 | 0.34 | 0.33 | 0.43 | 0.67 | 0.0149 | 0.3235 |
| Setdb2 | 1.25 | 1.24 | 1.28 | 1.03 | 0.0593 | 0.7680 |
| Setd2 | 1.04 | 0.47 | 1.06 | 0.37 | 0.0001 | 0.0007 |
| Setd3 | 1.23 | 1.40 | 1.45 | 1.71 | 0.0047 | 0.7636 |
| Setd4 | 0.49 | 0.91 | 0.56 | 0.71 | 0.0459 | 0.0095 |
| Setd5 | 0.60 | 0.85 | 0.88 | 1.82 | 0.0008 | 0.9074 |
| Setd7 | 1.23 | 1.37 | 1.09 | 1.48 | 0.5607 | 0.4297 |
| Setd8 | 0.54 | 0.35 | 0.43 | 0.30 | 0.0235 | 0.4246 |
| Suv39h1 | 0.56 | 0.50 | 0.65 | 0.57 | 0.9178 | 0.0267 |
| Suv39h2 | 0.53 | 0.40 | 0.62 | 1.34 | 0.0008 | 0.0644 |
| Prmt1 | 0.04 | 0.09 | 0.03 | 0.15 | 0.0004 | 0.0013 |
| Prmt2 | 0.51 | 0.51 | 0.39 | 0.55 | 0.8032 | 0.2393 |
| Prmt3 | 0.79 | 1.09 | 1.17 | 1.26 | 0.0020 | 0.6189 |
| Prmt5 | 0.32 | 0.23 | 0.19 | 0.27 | 0.3987 | 0.3265 |
| Prmt6 | 0.88 | 0.82 | 0.95 | 4.01 | 0.0012 | 0.7505 |
| Prmt7 | 0.33 | 0.31 | 0.30 | 0.34 | 0.9027 | 0.9004 |
| Prmt8 | 1.02 | 1.03 | 0.96 | 1.00 | 0.8367 | 0.4134 |
| Smyd2 | 1.60 | 0.86 | 0.95 | 0.84 | 0.0001 | 0.3807 |
| Smyd3 | 0.41 | 0.40 | 0.39 | 0.89 | 0.0059 | 0.9449 |
| Dnmt1 | 0.83 | 0.42 | 0.36 | 0.33 | 0.0003 | 0.5082 |
| Nsd1 | 0.95 | 1.02 | 1.01 | 1.45 | 0.0007 | 0.9227 |
| NSD2/Whsc1l1 | 0.52 | 0.90 | 1.33 | 1.90 | 0.0003 | 0.1770 |
| Idh1 | 0.54 | 0.57 | 1.03 | 1.62 | 0.0135 | 0.1516 |
| Idh2 | 0.15 | 0.24 | 0.15 | 0.45 | 0.0007 | 0.0610 |
| Eed | 0.27 | 0.24 | 0.44 | 0.67 | 0.0160 | 0.0909 |
| Aebp2 | 0.34 | 0.20 | 0.65 | 0.97 | 0.0072 | 0.0022 |
| Ezh1 | 0.28 | 0.27 | 0.29 | 0.45 | 0.0456 | 0.7687 |
| Ezh2 | 0.16 | 0.21 | 0.18 | 0.52 | 0.0008 | 0.6786 |
| Ehmt2 | 0.45 | 0.24 | 0.31 | 0.16 | 0.0002 | 0.2862 |
| Dyrk3 | 4.60 | 4.33 | 1.42 | 2.28 | 0.2615 | 0.0576 |
